# The Oxidative Stress and Inflammatory Metabolic Pathways of Some Environmental Toxicants Inflicting Human Disorders

**DOI:** 10.3390/toxics14090737

**Published:** 2026-08-22

**Authors:** Michael Brimacombe, David A. Lawrence

**Affiliations:** 1 Department of Pediatrics, University of Connecticut School of Medicine, Farmington, CT 06030, USA; 2 Connecticut Children’s Research Institute, Hartford, CT 06106, USA; 3 Departments of Biomedical Sciences and Environmental Health Sciences, College of Integrated Health Sciences, University at Albany, Albany, NY 12222, USA

**Keywords:** thiols, oxidative stress, inflammation, autoimmunity, aging

## Abstract

Numerous types of environmental toxicants alter human metabolomics, directly or indirectly, through gut microbial dysbiosis, upsetting immune homeostasis. Environmental toxicants capable of these direct and indirect effects have included heavy metals, pesticides, herbicides, polychlorinated biphenyl (PCB) congeners, and endocrine-disrupting chemicals. The cell and molecular events induced by some diverse toxicants are reviewed, along with their potential additive, synergistic, and antagonistic effects on immune homeostasis (increasing proinflammatory immune cell activation and suppressing immunoregulation), which leads to systemic oxidative stress (OS). As people are exposed in varying degrees to countless chemicals and environmental factors over a lifetime, it is challenging to correlate specific diagnoses to any single toxicant or exposure, which is often a key challenge in linking environmental exposure to health outcomes. However, many toxicants have the common mechanistic effect of OS. The effects of toxicants are more pronounced with aging due to cumulative exposures and immunoaging (immunosenescence), with chronic low-grade inflammation referred to as “inflammaging”. The cell and molecular mechanisms of toxicants include altered calcium flux, mitochondrial dysfunction, and increased levels of damage-associated molecular patterns (alarmins) that trigger an inflammatory response and possibly promote autoimmune and neurological disorders. OS skews type-1 immunity for defenses against pathogens and cancers more toward type-2 immune responses to self-antigens (autoimmunity). The toxicants may directly affect the innate and adaptive immune cells inducing this skewing, or they may modify portions of gut microbial species and strains and their production of metabolites that indirectly affect systemic immunity. These latter toxicant influences require metabolomic analysis of the differential structures and activities of the microbial metabolites. The damaging effects of OS and inflammation disrupting immune homeostasis and leading to disorders are reviewed and discussed. The need for well-designed studies that allow for standardized comparison of exposures and related effects are emphasized, and their real-world limitations noted.

## 1. Introduction

There tends to be a decline in protective immunity and an increase in autoimmune disease incidence with increasing advanced age, and exposure to environmental toxicants may accelerate aging-related mechanisms. The acquisition of differential immune changes with advanced age may be an intrinsic property of host innate and adaptive immune cells, as well as the cumulative effects of environmental toxicants on the immune cells and the microbiome-gut–brain axis (MGBA), which has regulatory effects on the cardiovascular, endocrine, and immune systems. An additional complexity is that toxic insults can arise from many types of toxic insults, including chemical, physical, and psychological stresses, e.g., in a mouse report, cold (physical)-restraint (psychological) stress inhibited immunity to a *Listeria monocytogenes* infection [1,2,3] in a manner similar to that of the heavy-metal effects of lead [4,5].

Additionally, Pb and psychological stress have been shown to generate additive, synergistic or potentiated effects [6]. Exogenous physical and psychological stresses may also relate to the increased prevalence of anxiety and depression in adolescents due to their induction of endogenous metabolites such as inflammasome products [7] and the kynurenine and serotonin pathways of tryptophan metabolism [8]. Although the decline of immune functions is associated with accumulated toxicant exposures and aging-related pathologies, including increased incidence of infectious diseases, autoimmune diseases, and cancers of the immune and other organ systems, it is likely that the cause of cellular and organism aging is due to genetics and lifelong environmental exposure histories, which affect cellular metabolism and regulatory inter-organ communications.

Certainly, toxic and infectious exposures and physical/psychological stresses can affect longevity, and immunosuppression as well as exaggerated, unregulated immune responses, including chronic inflammation, can shorten life expectancy; however, the particular cellular and molecular changes occurring with aging that lessen both quality of life and life span have not been fully delineated. This review focuses primarily on the cellular and molecular parameters of OS and the neuroendocrine immune (NEI) network.

**Figure 1:**
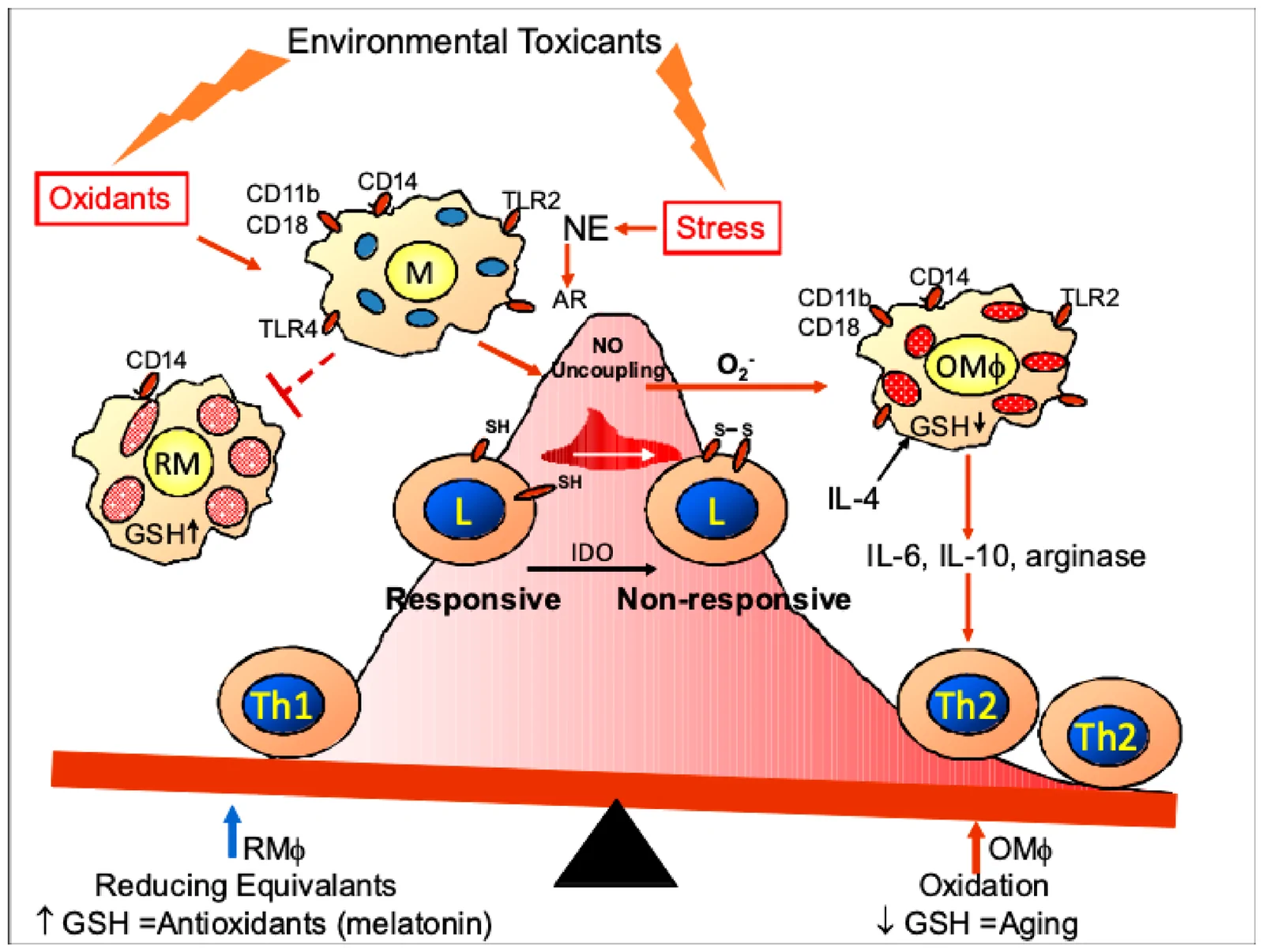
Environmental toxicants induce stress and oxidative metabolites that alter cellular thiols and skew immunity of type-1 helper T (Th1) cells to type-2 Th2 cells and antigen-presenting macrophages (RM or M1) with less (↓) glutathione (GSH) to OM or M2, which decreases cell-mediated immunity (CMI) responses to pathogens and cancers and enhances antibody-mediated immune (AMI)–autoimmune responses with interleukin (IL) -4, 6, and 10.

Environmental toxicants influence the thiols and functions of immune cells altering immunity via loss of reducing agents like glutathione (GSH) and the induction of oxidants and stress-related neurotransmitters. Figure 1 partially shows the inclusion of “NO uncoupling”, oxidants/antioxidants and the tryptophan–kynurenine pathway causing immunosuppression via indoleamine-2,3-dioxygenase (IDO) depletion of tryptophan. Environmental toxicant-induced stresses and oxidants skew and diminish immunity.

In general, the practical study of toxic and infectious exposures and related physiological stresses is challenging. In a human population, basic findings obtained in the study of mice or basic cell lines are often difficult to assess due to issues of study design, sample size, dosage, the accurate measurement of exposures and the timing of such exposures. The issue of causation versus correlation arises, and the accurate assessment of associated physiological stresses in the presence of mediating, secondary variables often require statistical models to determine significance. The importance of replication and adequate sample size, along with related statistical power, provide challenges for researchers. However, it is important to recognize that many different environmental chemicals have a common intermediate process of OS leading to MGBA metabolites with apparent linkages to immune disorders.

### 1.1. Methods and Materials

This narrative review of the literature was conducted using Google and PubMed sources. The set of keywords used were “environmental toxins and oxidative stress”, “Redox changes (Ox > Red) and aging”, “toxins” and “autoimmunity”. These were then organized by three general themes. These were the systemic effects of inflammation and aging, OS and immunity, and environmental stresses and redox effects on immune cells and health.

### 1.2. Inflammation and Aging

The cause of the decline of antigen-specific immunity is whether the immune dysfunction is due to the increased presence of inflammatory mediators in older persons due to age alone and/or whether inflammation is a consequence of age-related accumulation of toxic metabolites. Numerous changes in body composition and physiology have been noted with increasing age. Many diseases, including those associated with inflammatory processes, are age-related in terms of prevalence, but how these diseases relate to the primary aging processes remain to be defined, requiring investigation.

For example, the proinflammatory cytokine IL-6 has correlated positively with aging [9,10,11], and, upon infection of mice at higher bacterial burdens, the serum IL-6 concentration increases exponentially along with the stress-related hormone corticosterone, and the number of lymphocytes decline [12]; however, this is not evidence that IL-6 causes immune cell senescence or lowered responsiveness. The determination of causation requires careful study, an appropriate study design and a controlled experimental context.

Proinflammatory cytokines increase in centenarians (long-lived persons) [13,14] suggests that it may be the ability to cope with inflammation that is more critical than the ability to control inflammation. Since inflammation is closely associated with OS [15], it-is important to evaluate the mechanistic connections between inflammation, OS, and aging, because lymphocytes are highly sensitive to OS, and monocytes and neutrophils, which produce oxidants, are relatively resistant to OS. Additionally, innate immunity is intimately involved in inflammation. OS mechanisms influence the decline of antigen-specific immunity, including the availability of naïve T cells and their response to new antigens.

Innate immunity is designed to assist host defenses by rapidly lowering pathogen propagation and invasion prior to development of adaptive immunity. However, it is posited that the positive aspects of innate immunity decline in the elderly due to a shift toward the production of molecules such as IDO, which deplete the molecules necessary for enhanced immunity such as tryptophan (an amino acid needed for lymphocyte activity), and the production of reactive oxygen species (ROS) instead of nitric oxide (NO) (“the uncoupling of NO production”) [16], which leads to interference with T and B cell development and activation.

The availability of tetrahydrobiopterin (BH_4_), a co-factor for NO synthases and tyrosine and tryptophan hydroxylase, is linked with glutathione (GSH) levels and both decline due to the redox changes. Loss of BH_4_ is, in part, responsible for the uncoupling of NO and increased IDO activity, and BH_4_ loss is associated with cardiovascular diseases and neuropsychiatric disorders [17]. The age-associated decline in cellular energy with less ATP and a concomitant increase in intracellular oxidants (H_2_O_2_ and ˙NO) suggests that mitochondria malfunctioning may be central to the biology and chemistry of aging [18,19]. The connection of mitochondrial dysfunction as a key aspect of aging again implicates environmental toxins since many are known to affect mitochondrial functions [20].

### 1.3. OS and Immunity

Cellular thiol proteins influence membranal lipid domains, membranal lipids can influence the number of cellular thiols, and OS alters the lipid composition of the plasma membrane (PM) via lipid peroxidation [21], which can be initiated by secondary organic aerosol (SOA) toxicants [22]. Thus, depletion of cellular thiols can result in immunomodulation due to a restructuring of PM lipids and proteins (receptors and intracellular signal transduction circuits) of lymphocytes and antigen-presenting cells (APC), which can alter early signal transduction events (G0 G1) associated with APC.

The existence of protein–protein, protein–lipid and lipid–lipid domains within the PM and the influence of cellular thiols on these domains have been previously reported [23,24]. Interestingly, the rearrangement of membrane lipids after OS is often accompanied with extracellular expression of heat shock proteins (HSPs), which have immunomodulatory properties. HSP70 has been shown to increase Treg activity [25] and inhibit antigen presentation [26]. However, HSP70 also activates innate immune cells to release inflammatory cytokines [27]. Additionally, protein disulfide exchanges, which are affected by redox changes, can influence enzymatic activities associated with different immune activities, e.g., modifying the metalloprotease activity of TACE/ADAM-17, which affects the release of TNF, TNFR, and L-selectin [28,29]. However, as for the effects of HSP70, not all immune activities uniformly decline with the increased induction of stress proteins or increasing age [30]. CD8^+^ T cells tend to decline more rapidly than CD4^+^ T cells, and innate and humoral immunity (mainly B cell and Th2 cell activities) appears to outlast some other T cell functions [31].

In fact, enhanced innate cell induction of inflammation is, at least partially, responsible for the declining antigen-specific immunity. Additionally, macrophages with different amounts of GSH differentially activate lymphoid subsets, which produce different cytokines and immune responses [32,33,34,35,36]. Accumulated toxicants with age, and age itself, are posited to cause redox changes in the elderly, which involve an imbalance of NO and ROS, decline of BH_4_ and GSH, stress-induced norepinephrine, and lower oxidoreductase activity. Each of these changes are associated with the decline of antigen-specific immunity.

Numerous signaling events required for T cell activation are also thiol-modulated, including signaling via CD4 through Lck, phospholipase Cγ1 (PLCγl) and various kinases [37,38,39]. The conformation of the protein transmembrane domains of the TCR/CD3/CD4 or CD8 complex will likely affect their association with intra-membranal enzymes, cytoplasmic associates (e.g., Lck), and lipid–lipid domains. CD4 has intra- [39] and extracellular thiol influences on activation [40,41]. Likewise, the CD45 family [42] and L-selectin [43] have a known involvement in T cell activation via thiol changes. A change in membrane structure/fluidity also influences lymphocyte activation. Note that cholesterol, a known modulator of membrane structure, can modify antigen-specific presentation [44], and its availability is dependent on conditions known to vary with aging-related HDL/LDL changes and metabolic syndrome.

Numerous enzymes with immunomodulatory potential are thiol-regulated, including adenylate cyclase [45], 5-nucleotidase [46], insulin receptor [47], and Na+/K+ ATPase [48]. Changes in the lymphocytes’ transport functions start early in G1 (Na+, K+, and Ca++ changes occur within 1 min; glucose by 10 min; amino acids and nucleosides by 1 h). As cell transport mechanisms directly utilize thiol-sensitive proteins [49,50,51,52], thiol modulation has regulatory influence on multiple early events involved in lymphocyte activation. Na/K-ATPase activity decreases in lymphocytes from aged people [53]; membrane potential changes are less responsive with aged cells [54,55]; and nucleotide pools [56], kinase activity [57], protein phosphorylation patterns [58], and G protein involvements [59] have been reported to be modified by aging. Many of these processes are interconnected with the activation of T cells [60].

Previously published studies have shown that lymphocyte activation and proliferation are affected by cellular thiols (R-SH), which includes surface thiols [61,62,63] as well as total thiols [64] and GSH [65,66,67,68]. Polymorphisms of *GLCLC* and *GLCLR*, the two genes encoding glutamate cysteine ligase (GCL) [69], and the rate-limiting enzyme in GSH biosynthesis may be partially responsible for differential aging rates among the elderly. However, cellular thiols also influence the phospholipid and nucleotide composition of lymphocytes [68,70]. Since the toxic mechanism of action includes the ability to dysregulate the redox status, production of inflammatory mediators, epigenetic alterations, barrier deterioration, and alteration of mitochondrial function [71], the role of thiols classically fits into the modulatory scheme. These cellular, biochemical, and genetic modifications may lead to the development of autoimmune diseases [72].

Toxins dysregulate enzymatic processes due to cellular damage. Toxins themselves may bind to proteins to create new epitopes tricking the immune system into treating the body’s own constituents as foreign invaders. Examples are the dysregulated production of citrullinated proteins [73] and redox-mediated carbamylation [74] generating autoantibodies and immune complexes in the joints of rheumatoid arthritis patients. The citrullinated and carbamylation of self-proteins generate new epitopes, which allow breaking tolerance and the generation of autoantibodies. Note that an indicator of redox is the shortening of telomere length [75]. In addition to the Th1-Th2 imbalance, other innate and adaptive immune cells are involved in autoimmunity, for example Th17 and regulatory T cells (Treg cells) are influential in lupus [76]. The Th17 cells enhance inflammation and the loss of Treg cells further enhances the immune imbalance between CMI and AMI.

### 1.4. Environmental Stresses and Redox Effects on Immune Cells and Health

Chemical, physical, and psychological stressors are posited to cause an imbalance of the neuroendocrine immune (NEI) network, which then results in oxidative stress and inflammation; this imbalance occurs more frequently in older persons, who are, in general, more sensitive to these changes. Environmental stresses exacerbate age-related metabolic changes, including the lowered production of neurotransmitters and altered regulation of nitric oxide synthase (NOS) activity, which are intrinsic aspects of NEI mechanisms, and they cause increased production of reactive oxygen species and loss of cellular thiols [77]. These changes to redox status have been posited to lower antigen-specific immunity and to be implicated in age-related comorbidities. These comorbidities include cardiovascular disease, type-2 diabetes, and arthritis.

In a study with middle-aged mice [78], there was a decline in activation of nuclear factor erythroid 2-related factor 2 (Nrf2)-dependent antioxidant enzymes in lungs, liver and cerebellum in response to airborne nanoparticulate matter (nPM). The suppressed inducibility of the Nrf2-regulated antioxidant enzymes were glutamate cysteine ligase (both the GCLC and GCLM subunits), heme oxygenase 1 (HO-1) and NADPH:quinone oxidoreductase 1 (NQO1). The nPM effect was associated with an increased or exaggerated inflammatory response, which is suggested to play a central role in nPM-associated cardiopulmonary diseases [79,80,81,82]. NF-kB activation in response to particulates is observed in both human macrophages and NHBE epithelial cells [83,84]. The Nrf2-dependent antioxidant response is also a protective mechanism against oxidative stimuli in macrophages [85], including against particles [86].

Oxidative stress and aging have been linked to increased autoimmune activities, especially humoral (antibody-mediated) autoimmunity [87]. As indicated, B cells, in general, have greater thiol sensitivity than T cells (see Table 1) and [88], thus, it is surprising that B cell and Th2 cell activities appear to outlast some T cell functions [89], which emphasizes the need to investigate the molecular basis for aging and the oxidative sensitivities. The mechanisms involved in the differential activation of Th1 and Th2 cells with aging, HIV infection, or exposure to thiol-reactive chemicals [88] have not been fully delineated.

It has been reported that oxidative stress, mainly on APC, allows Th2 development to predominate. This outcome was suggested to be related mainly to the loss of IL-12 production; however, in addition to less IL-12 for Th1 cell activation, regulatory effects dependent on the ratio of macrophages with high (M1) or low (M2) levels of GSH, as described [34], need to be further considered regarding aging. This scheme is shown (see Figure 1) with the incorporation of the influence of stress and involvement of NO uncoupling. IL-4 is a cytokine promoting Th2 activities, which lowers the GSH level of macrophages [35], thus enhancing the number of M2 (oxMφ or alternatively activated) macrophages with less GSH. These GSH influences on Mφs further implicate thiol chemistry in immune modulations.

**Figure 2:**
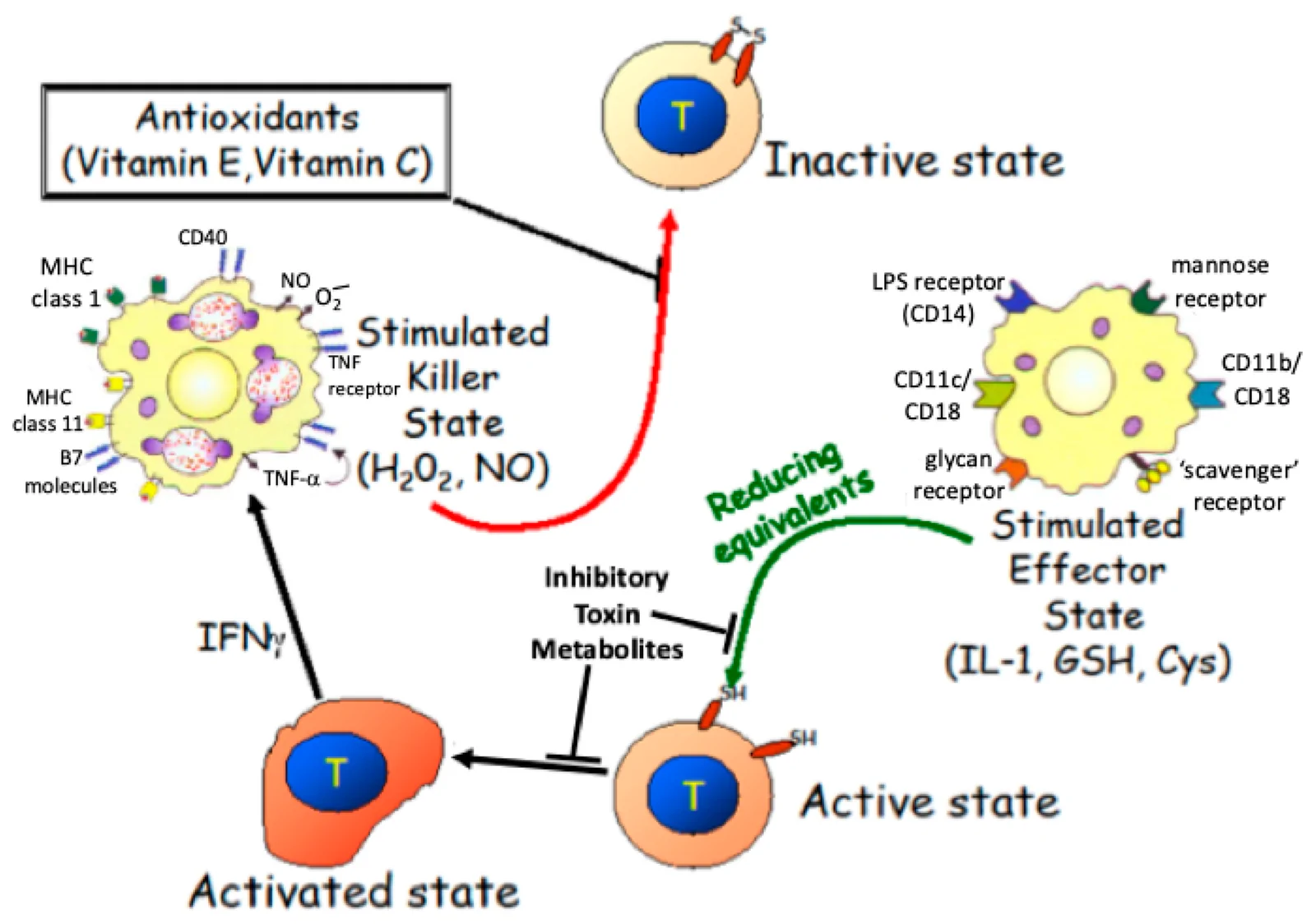
Macrophage redox regulation of T cells, which can be dysregulated by toxins.

Resting macrophages have been suggested to aid T cell proliferation via the release of proton donors, such as cysteine or GSH, whereas activated macrophages release more oxidants and are more inhibitory (Figure 2) and [90]. Toxic metabolites are shown to disrupt the redox influences of macrophages on T cells.

In general, increased exposures to environmental toxicants have become recognized by researchers, environmentalists, and physicians as major contributing factors to the rising prevalence of autoimmune diseases, asthma, and infections [91,92]. Many different toxicants are linked with OS influences on immunity leading to immune disorders [93,94,95,96,97,98]. An increase in cytosolic, and lipid reactive oxygen species (ROS), which is an iron-dependent form of programmed cell death referred to as ferroptosis, can also lead to autoimmune disorders [99]. A number of inflammatory disorders also occur due to the cyclic GMP-AMP synthase (cGAS) stimulator of the interferon genes (STING) signaling axis (cGAS -STING) which detects cytoplasmic nucleic acids and orchestrates innate immune responses against pathogens and endogenous DNA damage leading to inflammatory disorders [100,101].

### 1.5. Exposures, Context and Study Design Issues

The practical detection of various types of exposures, oxidative stress, and related immunological responses and toxicity is challenging. Many of the results reported here are based on carefully curated mouse cohorts or cell lines, studied in highly controlled experimental settings. To develop these results into a practical understanding relevant to the human population requires an appreciation of study design and related statistical modeling. The level of control possible in mouse and lab populations, and the level of genetic similarity and the careful control of exposures and length of exposures, is not typically possible in humans.

When developing a cohort or set of experimental subjects, ensuring they all share a similar environment, source of exposure and timing of exposure is very challenging and often results in a limited sample size and lower statistical power to detect defined effect sizes. Often, a case–control study is the most reasonable approach, beginning with a set of cases whose physiological condition and previous exposures are validated. To each case, a set of controls is identified that has similar exposure history but with no resulting physiological condition. Typically, a 2 to 1 or 3 to 1 ratio of controls to cases is taken. Methods for the analysis of case–control studies have been developed [102] and often rely on the detection of a significant odds ratio or relative risk when comparing cases versus controls. Cohort studies are also of relevance here [102].

Epidemiologic and environmental studies provide a context for a more general interpretation of results, but they are challenging to conduct as they often require baseline assessments of exposures and a follow-up based assessment of the onset of, for example, autism or infectious diseases. For illnesses examined in older populations, socio-economic bias, age cohort effects and underlying genetic susceptibility or immunity may affect the interpretation of the observed data and summary statistics such as rates, odds ratios and relative risks [102].

In the case of exposures to environmental risk factors, accumulated exposures and lifetime exposures, for example exposure to chemical agents which remain in the body, may also have an impact in regard to the onset of disease and yet are challenging to measure. There are more tools now to aid the linkage of different toxicants with immune disorders using AI and machine learning technologies; however, many influences of toxicants on the gut microbiota still have not been confirmed for immune disorders. Furthermore, the combinations of toxicant exposures may be better assessed by the level of cytokines, chemokines, and immune cell phenotypes.

## 2. Discussion

Oxidative stress and aging have been identified in basic research as affecting the immune system and other physiologic conditions, especially in regard to older individuals. Using these findings as a basis for (i) replication of these results in human subjects subject to various mediating variables and (ii) determining possible treatments or preventative practices in human populations is an ongoing challenge. To interpret results based on small sample sizes or, more generally, mouse and cell lines-based studies requires carefully designed studies.

If focusing on age cohorts within the human population, the need to identify and model mediating variables such as socio-economic effects, lifestyle, previous exposures and illnesses requires careful study design. This is challenging with limited resources. Further, the presence of genetic effects related to aging, the immune system, and sensitivity to toxic exposures are complex and may also play a role.

Many of the basic studies reported here were often limited in scope, identifying individual risk factors without consideration of a possible correlation with environmental or genetic factors when applying the results in human populations. Future studies taking these results and examining them in broader, controlled settings will be useful in extending the results to more practical settings.

## Figures and Tables

**Table 1:** Thiol/lipid characteristics of human lymphoid subsets.

Parameter Assessed	Relative Lymphoid Ranking
Radiosensitivity	CD19 > CD8 > CD4
Sensitivity to sulfhydryl-reactive compounds, e.g., N-ethylmaleimide	CD19 > CD8 > CD4
Sensitivity to oxidants, e.g., H_2_O_2_	B cells > T cells
GSH content	CD8 = CD4 > CD19
Exofacial thiols	CD19 > CD8 > CD4
Membrane fluidity	B cells > T cells
Ratio unsaturated: saturated lipids	B cells > T cells
Lipid packing (M540 insertion)	CD19 > CD8 > CD4
Associated methods and correlations between lipid composition, cellular thiols, and cellular sensitivities [88].	

## Data Availability

No new data were created or analyzed in this study. Data sharing is not applicable to this article.
